# Glial cell proteome using targeted quantitative methods for potential multi-diagnostic biomarkers

**DOI:** 10.1186/s12014-023-09432-x

**Published:** 2023-10-24

**Authors:** Narae Kang, Hyun Jeong Oh, Ji Hye Hong, Hyo Eun Moon, Yona Kim, Hyeon-Jeong Lee, Hophil Min, Hyeonji Park, Sang Hun Lee, Sun Ha Paek, Jonghwa Jin

**Affiliations:** 1 New Drug Development Center, Heungdeok-gu, Chungbuk, Cheongju-si, 28160 Korea; 2 Department of Neurosurgery, Cancer Research Institute and Ischemic/Hypoxic Disease Institute, Seoul National University, 28 Yeongeon-dong, Jongno-gu, Seoul, 03080 Korea; 3 Advanced Institute of Convergence Technology, Seoul National University (SNU), Suwon, 16229 Korea; 4 Department of Molecular Medicine & Biopharmaceutical Sciences, Graduate School of Convergence Science and Technology, Seoul National University, 28 Yeongeon-dong, Jongno-gu, Seoul, 03080 Korea; 5 Doping Control Center, Korea Institute of Science and Technology, Hwarang-ro 14-gil, Seongbuk-gu, Seoul, 02792 Korea; 6 School of Mechanical Engineering, Korea University, Seoul, 024841 Republic of Korea; 7 Institute of Chemical Engineering Convergence Systems, Korea University, Seoul, 02841 Republic of Korea; 8 Department of Chemical and Biological Engineering, Hanbat National University, Daejeon, 34158 Korea

**Keywords:** MRM, Glioblastoma, Primary cell, Biomarker, Quantitative proteomics

## Abstract

Glioblastoma is one of the most malignant primary brain cancer. Despite surgical resection with modern technology followed by chemo-radiation therapy with temozolomide, resistance to the treatment and recurrence is common due to its aggressive and infiltrating nature of the tumor with high proliferation index. The median survival time of the patients with glioblastomas is less than 15 months. Till now there has been no report of molecular target specific for glioblastomas. Early diagnosis and development of molecular target specific for glioblastomas are essential for longer survival of the patients with glioblastomas. Development of biomarkers specific for glioblastomas is most important for early diagnosis, estimation of the prognosis, and molecular target therapy of glioblastomas. To that end, in this study, we have conducted a comprehensive proteome study using primary cells and tissues from patients with glioblastoma. In the discovery stage, we have identified 7429 glioblastoma-specific proteins, where 476 proteins were quantitated using Tandem Mass Tag (TMT) method; 228 and 248 proteins showed up and down-regulated pattern, respectively. In the validation stage (20 selected target proteins), we developed quantitative targeted method (MRM: Multiple reaction monitoring) using stable isotope standards (SIS) peptide. In this study, five proteins (CCT3, PCMT1, TKT, TOMM34, UBA1) showed the significantly different protein levels (t-test: p value ≤ 0.05, AUC ≥ 0.7) between control and cancer groups and the result of multiplex assay using logistic regression showed the 5-marker panel showed better sensitivity (0.80 and 0.90), specificity (0.92 and 1.00), error rate (10 and 2%), and AUC value (0.94 and 0.98) than the best single marker (TOMM34) in primary cells and tissues, respectively. Although we acknowledge that the model requires further validation in a large sample size, the 5 protein marker panel can be used as baseline data for the discovery of novel biomarkers of the glioblastoma.

**Supplementary Information:**

The online version contains supplementary material available at 10.1186/s12014-023-09432-x.

## Statement of significance

For the discovery of multi-diagnostic biomarker, we have conducted a comprehensive proteome study using primary cells from patients with glioblastoma. In this study, 7429 glioblastoma-specific proteins were identified and then 20 selected target proteins were verified using MRM method. Finally, five proteins (CCT3, PCMT1, TKT, TOMM34, UBA1) showed the significantly different protein levels (t-test: p value ≤ 0.05, AUC ≥ 0.7) between control and cancer groups and the result of multiplex assay using logistic regression showed the 5-marker panel showed better sensitivity (0.80 and 0.90), specificity (0.92 and 1.00), error rate (10 and 2%), and AUC value (0.94 and 0.98) than the best single marker (TOMM34) in primary cells and tissues, respectively.

**Supplementary Information:**

The online version contains supplementary material available at 10.1186/s12014-023-09432-x.

## Introduction

Glioma is the most common primary cancer of the central nervous system, which is developed from glial cells and is generally classified into three subtypes such as Astrocytomas, Ependymomas, Oligodendrogliomas based on the type of glial cell involved in the tumor, as well as the tumor's genetic features [1, 2]. According to WHO guidelines (WHO 2016), glioma is classified into four grades (I-IV) and the most lethal grade is grade IV, glioblastoma multiforme (GBM) [2]; The incidence rate in the United States is 3.20 per 100,000 population [3], and GBM accounts for 60–70% of malignant gliomas [4]. GBM has only few patients reaching long- term survivor status and the median survival is 14.6 month and only 2.2% of patients are estimated to survive 3 years or more [5, 6].

The standard of care for GBM patients is surgical resection followed by adjuvant radiation therapy and chemotherapy with the temozolomide [5, 7]. Surgery provides ability to reduce the amount of solid tumor tissue within the brain and remove cells in the center of the tumor that may be resistant to radiation or chemotherapy. But Conventional therapies, have not resulted in major improvements in the survival outcomes of patients with glioblastoma [8–11].

The current diagnosis of the glioma is performed using the imaging techniques such as MRI [8, 9] or CT [10] and tissue biopsies [11]. These have some of limitations including the lack of accuracy of tumor position on brain and the difficulty of acquiring biopsies [12, 13]. For these reasons, glioma is harder to be diagnosed on early stage [14, 15]. The most of glioma patients have had a surgery followed by radiation therapy and chemotherapy, but it has not always shown excellent therapeutic effect. Therefore, discovering of early diagnosis and prognosis markers is very important for determination of appropriate treatment [16–19].

There have been many studies about analysis of correlation between GBM characteristics and specific molecular abnormalities for the past years [20]. Some cases showed the advancement in the pathogenic characterization of this disease [21–25]. So we need to better understand of which molecules are involved in disease manifestation and progression. In the past decade, differential proteomic profiling techniques have utilized tissue [22, 23], cerebrospinal fluid [23], and plasma [23–25] from glioma patients to identify the diagnostic, prognostic, predictive, and therapeutic response marker candidates, highlighting the potential for glioma biomarker discovery. The number of markers identified, however, have been limited, their reproducibility between studies is unclear, and none have been validated for clinical use [12].

Primary cell lines have been the historical standard both for the exploring the biology of human tumors in the preclinical models and for screening potential multi biomarker [26]. Primary cell lines reflect the tissue microenvironment, and it has no contamination unlike tissue [27]. Therefore, study of primary cell is that more can be effective approach to discovery of diagnostic and prognostic marker in the glioma than tissue.

In this study, we have performed comprehensive proteome analysis using the tandem mass tag (TMT) and targeted MS technique in the glioma patient-derived cell, glioma primary cell. We first applied integrated proteomic strategies to increase the depth of the primary cell proteome. Next, to validate the proteome expression using the MRM-MS method in primary cell with individual patients. Finally, to develop a multiplex assay, a multimarker panel was established, based on candidate variables in individual primary cells.

## Materials and methods

### Human surgical tissue samples and cell line

All fresh surgically resected tissue was diagnosed with glioblastoma according to WHO classification. Primary cells of human glioblastoma and astrocytes were obtained from brain tissue of the Brain Bank of Seoul National University Hospital. The mean age of controls and patients was 47.7 (31 ~ 68) and 56.28 (40 ~ 72), respectively (Additional file 1: Table S1). This study was approved by the Institutional Review Board (IRB) of Seoul National University Hospital (IRB approval H-0507-509-153).

### Cell culture and culture maintenance

Two cell lines were enzymatically dissociated to single cell from mechanically dissected glioblastoma and temporal lobe tissues. The cells were cultured in DMEM media (Welgene, Korea) supplemented with 10% fetal bovine serum (FBS; Gibco Invitrogen, USA), 100 U/mL of penicillin, and 100 mg/mL of streptomycin (Gibco Invitrogen) at 37 °C in an atmosphere of 5% CO_2_ in air. The cells were prepared from early passage less than 20 times and stocked (within 2 months) Additional file 2: Fig. S1).

### Protein digestion and TMT labeling

The cell pellets were prepared from control (5 samples) and cancer (5 samples) primary cells. They were collected in 15 mL falcon tubes, stored at − 80 °C until cell lysis was performed. Pellets were resuspended in lysis buffer; 8 M Urea, 10 × Protease inhibitor (cOmplete Protease Inhibitor Cocktail, Roche, Basel, Switzerland), 10 × Phosphatase inhibitor (phosSTOP, Roche, Basel, Switzerland) homogenized with a probe-type sonication (Marshall Scientific, Hampton, United States, 2 s 2 cycles 15% power) at 4 °C. The supernatants were move to new tube and measured protein concentration with BCA Protein assay (Pierce, Rockford, IL). Each 40 µg of proteins sample was reduced with 10 mM dithiothreitol (Sigma-Aldrich, St. Louis, Missouri, USA) and incubated for 30 min at 56 °C, followed by alkylation with 20 mM iodoacetamide (Sigma-Aldrich) and incubated for 30 min at dark room temperature. Trypsin-LysC (Promega, Madison, Wisconsin, United States) was added at a protein-to-enzyme ratio of 50:1 and samples were incubated overnight at 37 °C. For desalting, the enzymatic samples were clean-up using HLB oasis cartridge (Waters, Milford, Messachusetts, United States). The digests of the five cell lysates resuspended in 0.1 M TEAB (TEAB, sigma Aldrich, St. Louis, Missouri, United States) were labeled with five different Tandem Mass Tag (TMT, Thermo Scientific, Waltham, mesachusetts, United States) in anhydrous ACN according to manufacturer’s instructions. TMT labeled the five samples were collected in one tube and dried in vacuum.

### Mid pH reversed phase fractionation

TMT labeled peptides were subjected to mid-pH fractionation. Dried sample was reconstituted in 10 mM TEAB and loaded onto Agilent 1260 HPLC System (Agilent, Palo Alto, CA) equipped with fraction collector (set at 4 °C for all samples) coupled with a 4.6 × 250 mm XBridge C18 column (5 µm, 4.6 × 250 mm; Waters) with a flow rate of 0.4 mL/min. 10 mM TEAB pH 7.5 (Sol A) and 10 mM TEAB pH 7.5 in 90% ACN (Sol B) were used. Peptides were eluted with a gradient Sol B and collected into 96 well plate during 100 min. The separated samples were combined to 12 fractions and subsequently dried in speed vac. Peptides were recontituted in 0.1% formic acid water to analyze by LC–MS/MS.

### Protein identification by Q-Exactive analysis

For both identification and relative quantitation of GBM proteome, we were used Q-Exactive mass spectrometry coupled with an Easy-nLC 1000 (Thermo Fisher Scientific, San Jose, CA, USA). The extracted peptides were reconstituted in 0.1% formic acid and separated on EASY-Spray column (C18, 2 µm particle size, 75 µm X 500 mm). Samples were eluted from analytical column with a linear gradient of solvent B (100% ACN, 0.1% formic acid); 5–40% over 110 min, 40–80% over 7 min at a flow rate of 300 nL/min. The separated ions were moved into the mass spectrometer at an electrospray voltage of 2.1 kV. All MS/MS spectra were obtained in a data-dependent mode for fragmentation of the twenty most abundant peaks from the full MS scan with 32% normalized collision energy. The dynamic exclusion time was set at 30 s and the isolation window was 1.2 m/z. MS spectra were acquired with a mass range of 350–2000 m/z and 70,000 resolution at m/z 200. MS/MS resolution was acquired at a resolution of 17,500.

### Database searches and TMT labeled quantitation

Database searches (SEQUEST and X! Tandem) were performed using Proteome Discoverer (Thermo Fischer Scientific, ver 2.2.0.388) and Scaffold (version Scaffold_4.10.0, Proteome Software Inc., Portland, OR). Sequest and X! Tandem was set up to search a protein database, the uniprot-proteome_HomoSapiens_73099_FASTA. It was set by a fragment ion mass tolerance of 0.02 Da and a parent ion tolerance of 10.0 PPM. Carbamidomethyl of cysteine and TMT6 plex of lysine were specified in Sequest and X! Tandem as fixed modifications. Glu− > pyro-Glu of the n-terminus, ammonia-loss of the n-terminus, gln− > pyro-Glu of the n-terminus, oxidation of methionine and acetyl of the n-terminus were specified in X! Tandem as variable modifications. Oxidation of methionine and acetyl of the n-terminus were specified in Sequest as variable modifications. The Scaffold software (version 4.10.0, Proteome Software Inc., Portland, OR, USA) was used to validate MS/MS based peptide and protein identifications. Peptide identifications were accepted if they could be established at greater than 99.0% probability by the Scaffold Local FDR algorithm. Protein identifications were accepted if they could be established at greater than 5.0% probability to achieve an FDR less than 5.0% and contained at least 1 identified peptide.

Scaffold Q + (version 4.10.0) was used to quantitate Label Based Quantitation (TMT) peptide and protein identifications. Peptide identifications were accepted if they could be established at greater than 95.0% probability by the Scaffold Local FDR algorithm. Protein identifications were accepted if they could be established at greater than 5.0% probability to achieve an FDR less than 5.0% and contained at least 2 identified peptides. Protein probabilities were assigned by the Protein Prophet algorithm [28]. Proteins that contained similar peptides and could not be differentiated based on MS/MS analysis alone were grouped to satisfy the principles of parsimony. Proteins sharing significant peptide evidence were grouped into clusters. Of 395,679 spectra in the experiment at the given thresholds, 227,281 (57%) were included in quantitation. The normalized TMT signals were further analyzed by Perseus for statistical analysis. For each TMT experiment, the protein intensities were log2 transformed and subject to a median normalization. Significantly different protein levels between control and cancer groups for the three TMT experiments were calculated using a two-sided Student’s t-test using a permutation-based FDR cutoff (250 randomizations, FDR 0.01, S0 1). Proteins were considered as differentially regulated if their adjusted p-value corresponded to an FDR lower or equal to 0.01 and their fold change (expressed as log2 ratio) was < −2 or >  + 2.

### Gene ontology (GO) and functional analysis

The GO terms in the protein datasets were analyzed using the Scaffold bioinformatics resource (version 4.10.0), which performs functional classification and ID conversion of the proteins that we identified. The ‘biological process’, ‘molecular function’ and ‘cellular component’ classifications were analyzed using Uniprot accession numbers.

### Ingenuity pathway analysis

In order to further understand the biological significance of differentially expressed proteins, Ingenuity Pathway Analysis (IPA; Ingenuity® Systems, www.Ingenuity.com/) was used to analyze canonical pathways and biomarker filter. The proteomic data set included fold changes of protein was submitted into Ingenuity Pathway Analysis for core analysis, protein interactions regulated pathway analysis.

The core analysis was carried out with the settings of indirect and direct relationships between molecules which is come from our experimental data and data sources of the Ingenuity Knowledge Base. The probability that show the relationship of biological functions and diseases in the protein dataset is represented by the Right-tailed Fisher’s exact test.

### Synthetic peptides

For the MRM analysis, we first synthesized crude SIS (stable isotope-labeled standard) peptides for target peptides. Synthetic peptides were obtained from JPT Peptide (JPT Technologies, Berlin, Germany). Peptide sequences were synthesized as unmodified peptides with free N- and C-terminal amino acids. If there was carbamoylmethylation on a cysteine, the peptide was synthesized as the “carbamoylmethylation” form. For stable isotope-labeled peptides (heavy peptide), the C-terminal arginine or lysine contained ^13^C- and ^15^N-labeled atoms.

### Multiple reaction monitoring using triple quadrupole mass spectrometry

For the MRM (multiple reaction monitoring) analysis, digested peptides were analyzed by online nanoflow LC–MS/MS on a NanoAcquity UPLC system (Waters) that was connected to a 6500 QTRAP (AB Sciex, Framingham, MA) through a nanoelectrospray ion source. Briefly, digested peptides were loaded at a flow rate of 300nL/min by an autosampler onto a precolumn (2 cm long; ID, 180 μm; particle size, 5 μm) and an analytical column (10 cm long; ID, 150 μm; particle size, 1.7 μm), which were both packed with reversed-phase C18 material. The peptides were separated on a linear ACN gradient from 5 to 35% for 70 min and from 35 to 70% for 20 min, and peptides were eluted between 3 and 70 min. The optimal parameters for the triple quadrupole mass spectrometer that was interfaced with a nanospray source were as follows: ion spray (IS), 2300 V; source temperature, 160 °C; high collision gas, approximately 4 ~ 3 × 10^–5^ torr; and curtain gas, 20. MS parameters for declustering potential (DP) and collision energy (CE) were determined using the Skyline program. In the MRM run, the scan time for each transition were set to 20 ms respectively.

The MRM assay was optimized with Skyline v20.2 (MacCoss Lab). Transitions which have high peak intensity—all possible b- and y-ion series—were chosen for each peptide at a 2/3 + charge state. The best 1 transition was selected for further analysis, and CEs were optimized for each transition. The energy was ramped around the predicted value per the default formula (CE = 0.057x–4.262) in 5 steps on both sides with 2-V increments, and the best CE energy was selected, based on the optimal signal intensity, as manually assessed.

### Statistical analysis

To develop a reliable classifier from differentially expressed proteins, we used SPSS (Armonk, NY: IBM Corp., version 26) to perform t-test and chi-square tests and generate receiver operating characteristic (ROC) curves. Medicalc (MedCalc Software, Mariakerke, Belgium) was also used for construction and evaluation of multi-marker panel and survival analysis was also performed using Kaplan–Meier method.

## Results

### Study layout for developing biomarker candidates from primary cell to diagnose the Glioma

**Fig. 1:**
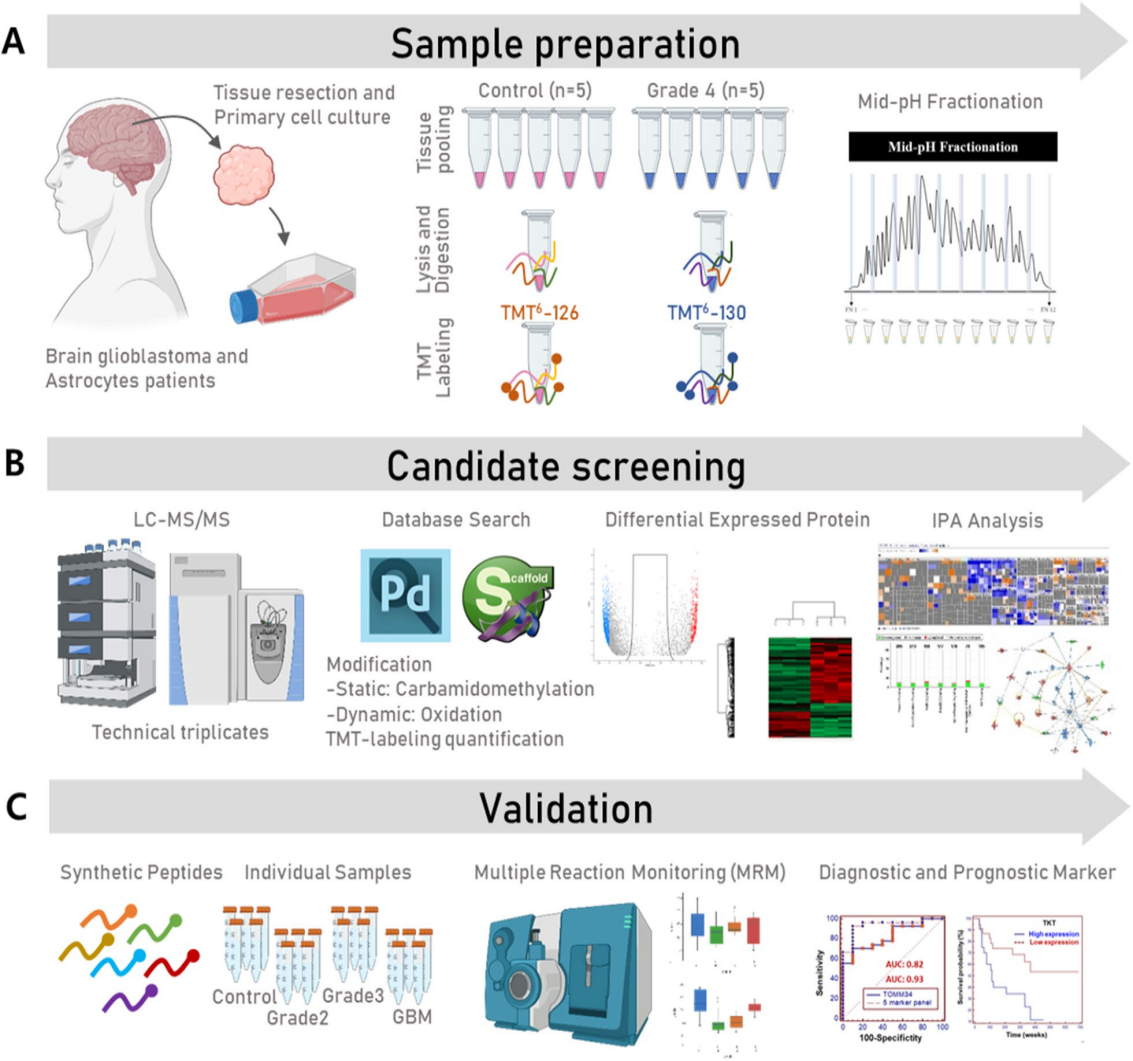
Schematic describing the Glioma primary cell biomarker Study workflow. Sample preparation **A**: surgically-removed tissue samples were enzymatically dissociated to single cells and cultured. Control pooled primary cells and grade 4 glioma pooled primary cells were lysed, digested, and labeled with TMT reagent 126 and 130, respectively. TMT-labeled control and grade 4 glioma peptides were mixed and subjected to HPLC fractionation. Candidate screening **B**: Fractionations obtained (n = 12) were subjected to LC–MS/MS, and the acquired data were analyzed via Proteome Discoverer to obtain differentially expressed proteins in glioma primary cells. Ingenuity pathway analysis (IPA) were performed to further understand the biological significance of the differentially expressed proteins. Validation **C**: MRM assays for the differentially expressed proteins were developed using synthetic peptides for each protein

The first step in biomarker development is to identify candidates. To this end, we performed a comprehensive proteomics study of glial cells, which pooled glial primary cells (Control: 5 and Grade 4: 5) were used. Next step is to validate the glial marker candidates in primary glial cells and tissues (Control: 10 and 10, Grade 2: 10 and 10, Grade 3: 12 and 10, and Grade 4: 15 and 10) (Fig. 1 and Additional file 1: Table S1). Briefly, in the first stage, we profiled the human glial proteome to obtain a pool of biomarker candidates, in which TMT-labeled quantitation method was performed to compare the abundance of proteins between control and cancer. Then, we stratified biomarker candidates by small scale MRM analysis, which was used as the initial selection tool in our systematic proteomic pipeline. In the second stage, a large-scale MRM analysis of targeted peptides was performed in individual glial cells and tissues using the corresponding heavy peptide mixtures as an internal standard. Finally, to develop a multiplex assay, a multimarker panel was established, based on candidate variables in individual primary cells.

### Identification of protein in glial primary cell

To obtain an in-depth proteome in glial primary cell, we implemented the TMT labeling method combined with LC-based mid pH peptide fractionation. Our proteome analysis was performed based on the high-resolution mass spectrometry and a multiple-database search strategy including SEQUEST and X! Tandem. In this study, a total of 7,429 protein groups were identified at a minimum confidence level > 95%, more than 2 unique peptides, and FDR < 5% (Additional file 1: Table S2).

To determine the functions of the proteins in our glial proteome, we used Gene Ontology (GO) to classify them by biological process (BP), molecular function (MF), and cellular component (CC).

Our glial proteome was significantly enriched in proteins that participate in ‘cellular process (38.6%), ‘biological regulation’ (29.4%), and ‘metabolic process’ (25.8%). Regarding molecular function, the proteome was significantly enriched in proteins that mediate ‘binding’ (34.4%), ‘catalytic activity’ (17.7%), and ‘enzyme regulator activity’ (3.3%). GO analysis of cellular components was significantly enriched in proteins associated with ‘intracellular organelle’ (37.2%), ‘cytoplasm’ (35.7%), and ‘membrane’ (21.8%) (Additional file 2: Fig. S2).

### Differential expression of proteins in control and glioma cell

For the differential proteome in control and cancer cells, three technical replicates were performed, and the labeled TMT quantitation method was used to compare protein expression under different conditions.

**Fig. 2:**
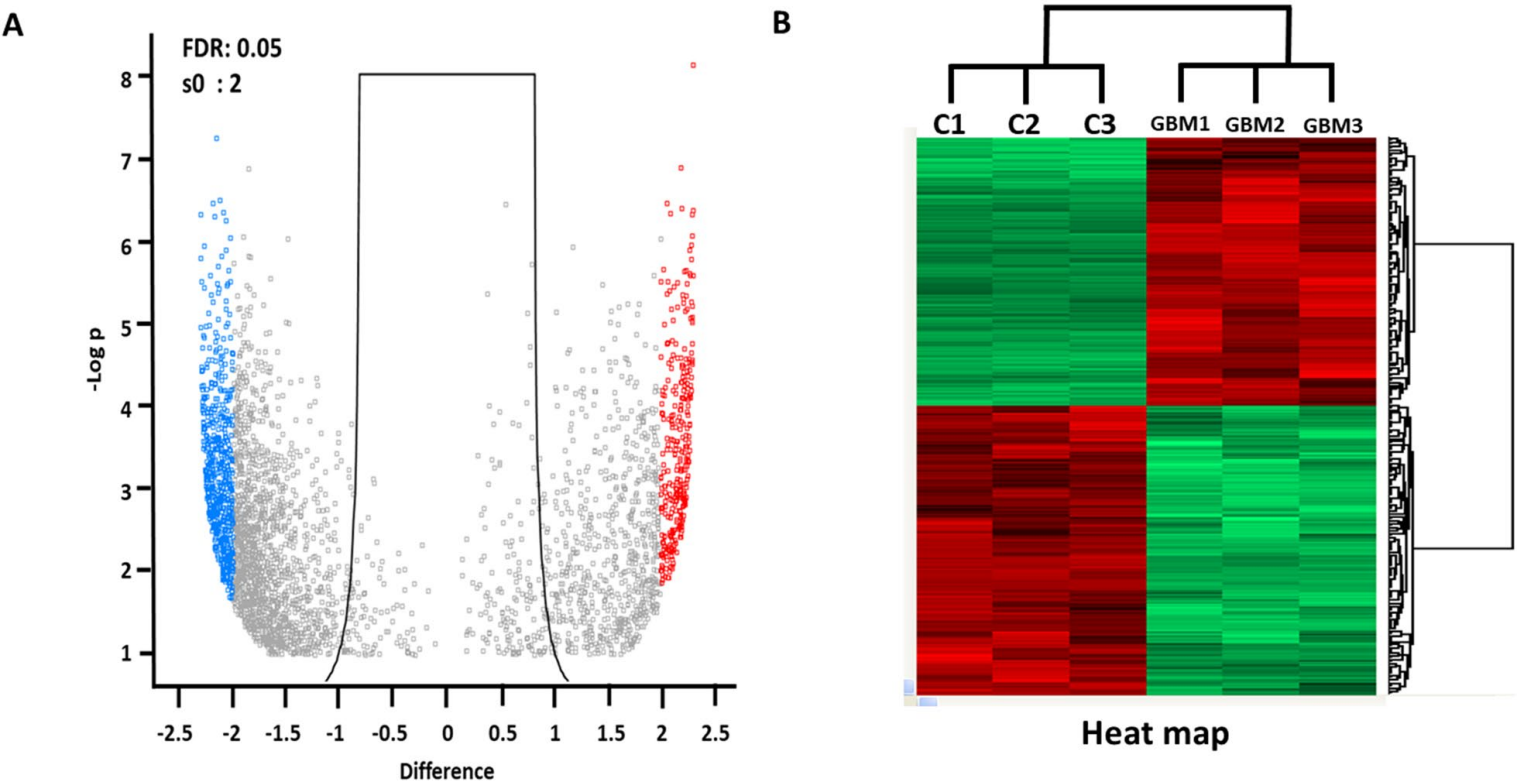
Differentially expressed proteins. The cutoff range of protein identification is as follows: protein confidence interval > 95.0%, peptide N ≥ at least two peptides, 1% < decoy FDR. Through t-test, p ≤ 0.05, and cv < 20%, total 476 proteins were finally listed as differentially expressed proteins. Volcano plot (A): For the analysis of differentially expressed proteins and statistical analysis, Perseus (version 1.5.8.2) and R were used, where the cutoff range for significant fold change (FC) and T-test p-values were set as ± 2.0 and 0.05, respectively. Heat map (B): 2D–hierarchical clustering analysis exploring the difference in protein expression between Red and Green Pashmina fiber. Each row in the map represents a differentially expressed proteins, and each column represents the condition used. Log_2_ (DEP) value was used for constructing the heat-map

To identify reliable key proteins that are systemically able to show the differentially expressed pattern, we first narrowed down the proteins based on the cutoff range rule (t-test, p value ≤ 0.05, minimum confidence level > 95%, more than 2 unique peptides, and FDR < 1%), and selected 3,311 proteins. We then determined the fold-change thresholds (expressed as log2 ratio) of > 2 or < -2 to identify true differences in the expression of proteins. Finally, to select a more reliable list of differentially expressed proteins, we assessed the technical variability based on the coefficient of variation (CV) in all experiments (CV < 20%). Four hundred and seventy-six proteins were finally quantifiable based on the above quantitative criteria (Fig. 2 and Additional file 1: Table S3), and these differentially expressed proteins were represented by volcano plots and heat maps (Fig. 2). Notably, three replicate experiments in control and glioma samples were used to show experimental accuracy and reproducibility.

### Analysis of canonical pathway and protein networks

To investigate the signaling pathway and protein–protein interactions related to the upregulated and downregulated proteins in our glial proteome, we performed canonical pathway and protein network analyses based on the differentially expressed proteins using IPA. Compared with control samples, there were 476 differentially expressed proteins in grade 4 glioma, of which 228 proteins increased, whereas 248 proteins decreased in abundance. In the canonical pathway, 476 regulated proteins were enriched in 470 pathways, where 21 representative signaling pathways related the carcinogenesis and neurogenesis are as followed; Protein Ubiquitination, Protein Kinase A Signaling, Sertoli Cell-Sertoli Cell Junction Signaling, PI3K/AKT Signaling, Leukocyte Extravasation Signaling, Systemic Lupus Erythematosus Signaling, IGF-1 Signaling, 14–3-3-mediated Signaling, HIPPO signaling, ERK5 Signaling, Inhibition of ARE-Mediated mRNA Degradation, Necroptosis Signaling, Calcium Signaling, FAT10 Signaling, Mitochondrial Dysfunction, Cell Cycle, Pentose Phosphate, DNA Methylation and Transcriptional Repression Signaling, Apelin Adipocyte Signaling, Neuroprotective Role of THOP1, and TCA Cycle II pathway (Table 1).

**Table 1:** Analysis of canonical pathway and protein networks

N	Ingenuity Canonical Pathways	-log (p-value)	Ratio	z-score	Down regulated %	No change %	Up regulated	No overlap with dataset %	Gene name
1	Protein ubiquitination pathway	5.82	0.0784	–	4/268 (1)	0/268 (0)	17/268 (6)	247/268 (92)	DNAJB1,DNAJC2,HSPB6,HSPB8,HSPD1,HSPE1,HSPH1,PSMC1,PSMC2,PSMC4,PSMD1,PSMD11,PSMD12,PSMD3,PSME1,SUGT1,THOP1,UBA1,UBE2O,USP24,USP8
2	Protein kinase A signaling	2.71	0.0494	0.243	14/385 (4)	0/385 (0)	5/385 (1)	366/385 (95)	ADD3,CALM1,FLNA,GNG12,GNG2,GYS1,H10,ITPR3,MAP2K1,PLCB3,PPP1R10,PPP1R12A,PPP1R14A,PPP1R3D,PTPN12,PTPRJ,ROCK2,TGFB2,YWHAE
3	Sertoli cell-sertoli cell junction signaling	5.23	0.0884	–	14/181 (8)	0/181 (0)	2/181 (1)	165/181 (91)	ACTB,ACTN1,AKT1,AKT3,ILK,ITGB1,JAM2,JAM3,KRAS,MAP2K1,MAP2K3,MPP6,RALA,RRAS,TJP1,VCL
4	PI3K/AKT signaling	4.23	0.0809	− 1.387	10/173 (6)	0/173 (0)	4/173 (2)	159/173 (92)	AKT1,AKT3,GRB2,GYS1,ILK,INPP5K,ITGB1,KRAS,LIMS1,MAP2K1,RALA,RRAS,SYNJ2,YWHAE
5	Leukocyte extravasation signaling	2.69	0.0622	0	9/193 (5)	0/193 (0)	3/193 (2)	181/193 (94)	ACTB,ACTN1,ARHGAP35,CD44,CD99,ITGB1,JAM2,JAM3,ROCK2,THY1,VCL,WASL
6	Systemic lupus erythematosus In T cell signaling pathway	2.3	0.0556	0	8/216 (4)	0/216 (0)	4/216 (2)	204/216 (94)	AKT1,AKT3,CD44,DNMT1,GRB2,KRAS,MAP2K1,MAP2K3,RALA,RND3,ROCK2,RRAS
7	IGF-1 signaling	4.49	0.106	− 0.707	7/104 (7)	0/104 (0)	4/104 (4)	93/104 (89)	AKT1,AKT3,CCN1,CCN2,GRB10,GRB2,KRAS,MAP2K1,RALA,RRAS,YWHAE
8	14–3-3-mediated signaling	2.54	0.0714	− 1	6/126 (5)	0/126 (0)	3/126 (2)	117/126 (93)	AKT1,AKT3,GRB2,KRAS,MAP2K1,PLCB3,RALA,RRAS,YWHAE
9	HIPPO signaling	2.45	0.0833	–	4/84 (5)	0/84 (0)	3/84 (4)	77/84 (92)	CD44,PPP1R10,PPP1R12A,PPP1R14A,PPP1R3D,TP53BP2,YWHAE
10	ERK5 Signaling	2.17	0.0833	0	3/72 (4)	0/72 (0)	3/72 (4)	66/72 (92)	AKT1,KRAS,RALA,RPS6KA3,RRAS,YWHAE
11	Inhibition of ARE-mediated mRNA degradation pathway	0.801	0.041	− 1.342	3/122 (2)	0/122 (0)	2/122 (2)	117/122 (96)	AKT1,AKT3,DDX6,PSME1,YWHAE
12	Necroptosis signaling pathway	0.541	0.0327	1.342	1/153 (1)	0/153 (0)	4/153 (3)	148/153 (97)	PLA2G4A,STAT1,TIMM13,TIMM8A,TOMM34
13	Calcium signaling	0.305	0.0253	− 1	3/198 (2)	0/198 (0)	2/198 (1)	193/198 (97)	ATP2B4,CACNA2D1,CALM1 (includes others),ITPR3,MYH14
14	FAT10 signaling pathway	3.16	0.222	–	1/18 (6)	0/18 (0)	3/18 (17)	14/18 (78)	PSME1,SQSTM1,UBA1,UBA6
15	mitochondrial dysfunction	0.267	0.0242	–	2/165 (1)	0/165 (0)	2/165 (1)	161/165 (98)	ATP5ME,CPT1A,GSR,NDUFAB1
16	pentose phosphate pathway	1.66	0.2	–	1/10 (10)	0/10 (0)	1/10 (10)	8/10 (80)	PGD,TKT
17	DNA methylation and transcriptional repression signaling	0.724	0.0588	–	1/34 (3)	0/34 (0)	1/34 (3)	32/34 (94)	DNMT1,H4-16
18	Apelin adipocyte signaling pathway	0.256	0.0253	–	1/79 (1)	0/79 (0)	1/79 (1)	77/79 (97)	GNA11,PRDX6
19	Neuroprotective role of THOP1 in Alzheimer's disease	0	0.0183	–	0/109 (0)	0/109 (0)	2/109 (2)	107/109 (98)	THOP1,YWHAE
20	TCA Cycle II (Eukaryotic)	0.363	0.0417	–	0/24 (0)	0/24 (0)	1/24 (4)	23/24 (96)	IDH3A
21	Cell Cycle: G2/M DNA damage checkpoint regulation	0.971	0.0612	–	0/49 (0)	0/49 (0)	3/49 (6)	46/49 (94)	TGFB2

### Validation of biomarker candidates in the MRM analysis

To select biomarker candidates, we first excluded proteins that have common gene and protein names. For the selection of reliable MRM transition, we constructed a glial-specific MS/MS spectral library and compared its MS/MS spectra with experimental spectra from our MRM analysis. In this study, 321 proteins showing the same fragmentation spectral pattern were selected. We then examined the detectability of marker candidates in the MRM platform, and confirmed low, middle, and high endogenous concentrations of the marker candidates, wherein the ranges of low, middle, and high concentrations were defined by comparing endogenous peptides with heavy peptide concentrations. For the MRM validation analysis, we excluded proteins with no detected range of concentration. To narrow down the number of marker candidates, we performed a small-scale MRM analysis, wherein we verified whether candidates showed the same expression pattern between the MRM and TMT-labeled dataset. From the small-scale MRM, 90 proteins were selected. Finally, bioinformatics analysis of the differentially expressed proteins revealed several putative enriched functional and disease networks, and upstream regulators, such as cancer, cell death and survival, organismal injury, and abnormalities related networks, which were used to select biomarker candidates. Consequently, 20 proteins, viz., ATP2B4, ATP5ME, CCT3, DNMT1, FKBP2, GLRX5, IDH3A, JAM2, LDHA, PCMT1, PLEKHG3, PRDX6, SLC44A2, TACC3, TINAGL, TKT, TOMM34, UACA, UBA1, and YWHAE were selected (Table 2).

**Table 2:** Selected 20 proteins and MRM transition

No	Gene name	Peptide sequence^a^	Q1^b^ (Da)	Q3^c^ (Da)	Charge^d^	MRM (T-test)^e^			MRM (AUC)^f^		
						(C vs G2)	(C vs G4)	(C vs G3 and G4)	(C vs G2)	(C vs G4)	(C vs G3 and G4)
1	ATP2B4	Light EGDFGCTVMELR Heavy EGDFGCTVMEL[13C-15N-R]	Light 678.8 Heavy 683.804	Light 908.433 Heavy 918.441	2 +	0.524129	0.464897	0.87613	0.500	0.513	0.441
2	ATP5ME	Light ELAEDDSILK Heavy ELAEDDSIL[13C-15N-K]	Light 566.79 Heavy 570.797	Light 690.367 Heavy 698.381	2 +	0.158475	0.407572	0.090901	0.340	0.387	0.293
3	CCT3	Light AVAQALEVIPR Heavy AVAQALEVIP[13C-15N-R]	Light 583.848 Heavy 88.852	Light 797.488 Heavy 807.496	2 +	0.4499	0.004443	0.006446	0.600	0.867	0.800
4	DNMT1	Light VARPLPAEEPER Heavy VARPLPAEEPE[13C-15N-R]	Light 682.37 Heavy 87.374	Light 1037.526 Heavy 1047.534	2 +	0.640442	0.069085	0.243172	0.650	0.853	0.689
5	FKBP2	Light LVIPSELGYGER Heavy LVIPSELGYGE[13C-15N-R]	Light 666.862 Heavy 671.866	Light 1007.479 Heavy 1017.488	2 +	0.282686	0.511163	0.46159	0.570	0.587	0.578
6	GLRX5	Light DYAAYNVLDDPELR Heavy DYAAYNVLDDPEL[13C-15N-R]	Light 827.391 Heavy 832.396	Light 1070.548 Heavy1080.556	2 +	0.523981	0.800963	0.171209	0.370	0.480	0.304
7	IDH3A	Light WMIPSEAK Heavy WMIPSEA[13C-15N-K]	Light 481.244 Heavy 485.251	Light 531.277 Heavy 539.292	2 +	0.616804	0.060986	0.294098	0.420	0.687	0.556
8	JAM2	Light ATTMSENDFK Heavy ATTMSENDF[13C-15N-K]	Light 572.253 Heavy 576.26	Light 739.326 Heavy 747.34	2 +	0.336735	0.072686	0.171878	0.580	0.707	0.644
9	LDHA	Light VTLTSEEEAR Heavy VTLTSEEEA[13C-15N-R]	Light 567.786 Heavy 572.79	Light 821.364 Heavy 831.372	2 +	0.098785	0.562608	0.087983	0.710	0.420	0.296
10	PCMT1	Light: VFEVMLATDR Heavy: VFEVMLATD[13C-15N-R]	Light 590.805 Heavy 595.809	Light 934.466 Heavy 944.474	2 +	0.580865	0.004685	0.317842	0.510	0.833	0.600
11	PLEKHG3	Light SIVEDYLLK Heavy SIVEDYLL[13C-15N-K]	Light 540.303 Heavy 544.31	Light 879.482 Heavy 887.496	2 +	0.236567	0.025892	0.440049	0.590	0.227	0.407
12	PRDX6	Light LPFPIIDDR Heavy LPFPIIDD[13C-15N-R]	Light 543.303 Heavy548.307	Light 728.394 Heavy 738.402	2 +	0.187465	0.263135	0.450441	0.310	0.593	0.411
13	SLC44A2	Light YLTYLNAR Heavy YLTYLNA[13C-15N-R]	Light 507.274 Heavy 512.279	Light 737.394 Heavy 747.402	2 +	0.552204	0.106011	0.376704	0.570	0.680	0.659
14	TACC3	Light AQAEALALQASLR Heavy AQAEALALQASL[13C-15N-R]	Light 671.378 Heavy 676.382	Light 1142.653 Heavy 1152.661	2 +	0.452203	0.183185	0.402999	0.450	0.707	0.637
15	TINAGL1	Light ITGWGEETLPDGR Heavy ITGWGEETLPDG[13C-15N-R]	Light 715.849 Heavy 720.853	Light 444.22 Heavy 454.228	2 +	0.232988	0.935397	0.507493	0.540	0.267	0.219
16	TKT	Light AYGQALAK Heavy AYGQALA[13C-15N-K]	Light 411.229 Heavy 415.237	Light 587.351 Heavy 595.365	2 +	0.522284	0.000576	0.293373	0.390	0.873	0.644
17	TOMM34	Light AAGNESFR Heavy AAGNESF[13C-15N-R]	Light 426.204 Heavy 431.208	Light 709.326 Heavy 719.335	2 +	0.044977	0.013853	0.019581	0.840	0.880	0.826
18	UACA	Light YAPIVSFEECER Heavy YAPIVSFEECE[13C-15N-R]	Light 750.345 Heavy 755.35	Light 956.378 Heavy 966.386	2 +	0.4204	0.48008	0.06091	0.620	0.433	0.281
19	UBA1	Light GLGVEIAK Heavy GLGVEIA[13C-15N-K]	Light 393.74 Heavy 397.747	Light 616.366 Heavy 624.381	2 +	0.944065	0.018036	0.153785	0.460	0.747	0.600
20	YWHAE	Light YDEMVESMK Heavy YDEMVESM[13C-15N-K]	Light 566.238 Heavy 570.246	Light 853.379 Heavy 861.394	2 +	0.44896	0.218744	0.207092	0.390	0.620	0.607

^a^Sequence represents the sequence of proteotypic peptide for target protein

^b–c^Q1 and Q3 (*m/z*) represent the Q1 and Q3 transitions for proteotypic peptide, respectively

^d^Charge represents the charge state of precursor ion

^e^T-test and

^f^AUC represent the p-value and area under the curve values in MRM, where C, G2, G3, and G4 are control, grade 2, grade 3, and grade4, respectively

### Individual sample analysis by MRM

**Fig. 3:**
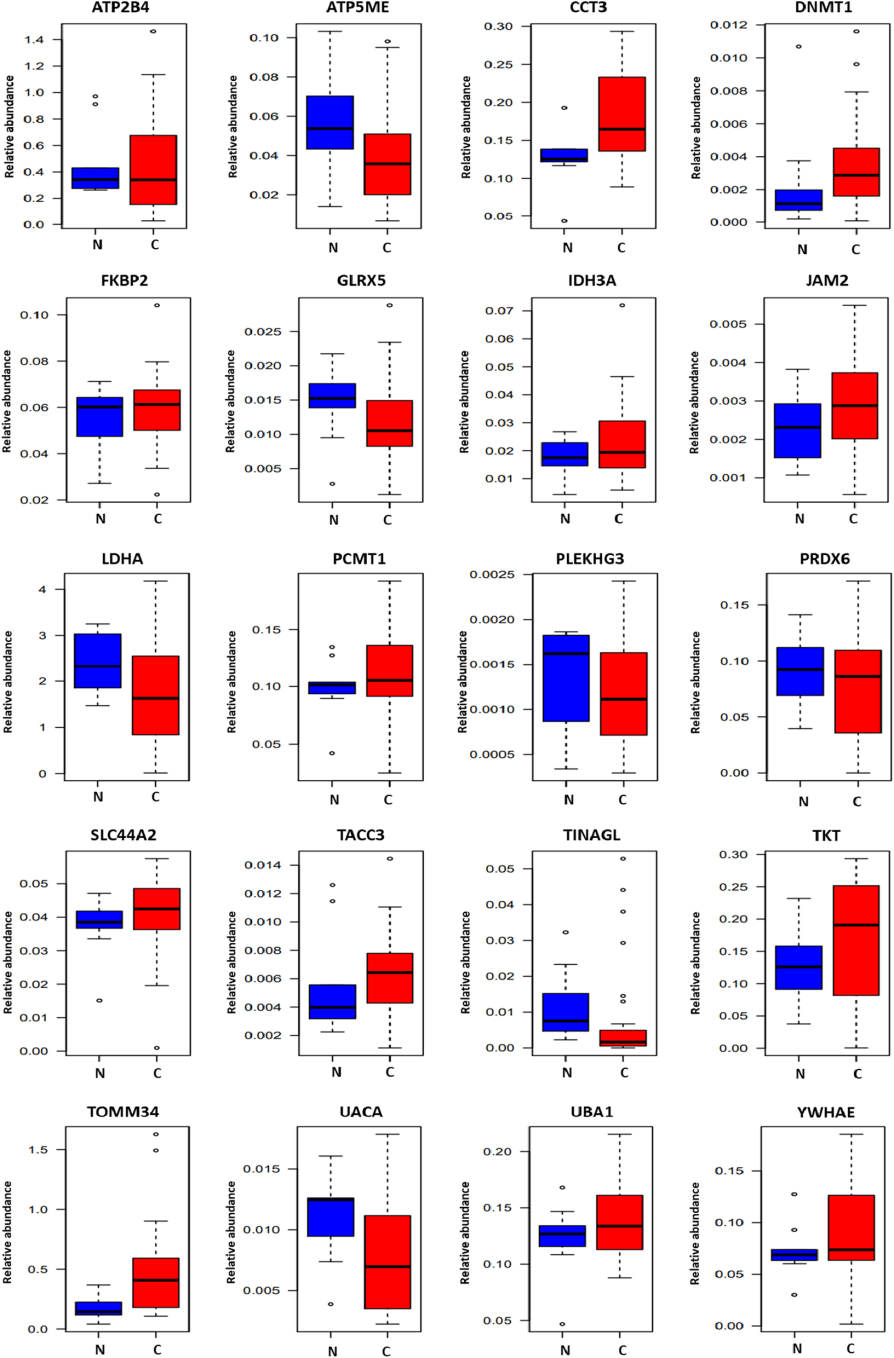
Validation of biomarker candidates in control group and cancer group. The 20 selected proteins from TMT labeled quantitation were verified by MRM in control (N = 10) and cancer (grade 3& grade 4) (N = 27) primary cell samples

Using the heavy peptide mixture (20 fmol/μL) of each target peptide for MRM as an internal standard, we analyzed individual human primary cells by MRM. We first confirmed the differential concentration of target proteins between control (N: 10) and cancer (grade 2: 10, grade 3: 12, grade 4: 15). All 20 proteins were detected in glial cells, and 5 proteins had disparate expression patterns in the control and cancer groups (Fig. 3 and Table 2). Student’s t-test and ROC curve was performed to compare the control and cancer groups; 5 (CCT3, PCMT1, TKT, TOMM34, UBA1) and 2 proteins (CCT3 and TOMM34) were satisfied with the significant differences rule (Student’s t-test: p ≤ 0.05, AUC: AUC value ≥ 0.7) in control versus cancer (grade 4) and control versus cancer (grade 3and 4), respectively.

### Construction of a multi-marker panel based on the MRM results

To improve the classification discriminating power between the control and cancer groups, we constructed a multi-marker panel using Logistic regression analysis and used it to statistical evaluation.

We first selected a multi-marker panel that showed the best discriminatory power between the control and cancer group (grade 4). We then applied this multi-marker panel to evaluate its discriminatory power in control group versus cancer group (grade 3 and 4).

**Fig. 4:**
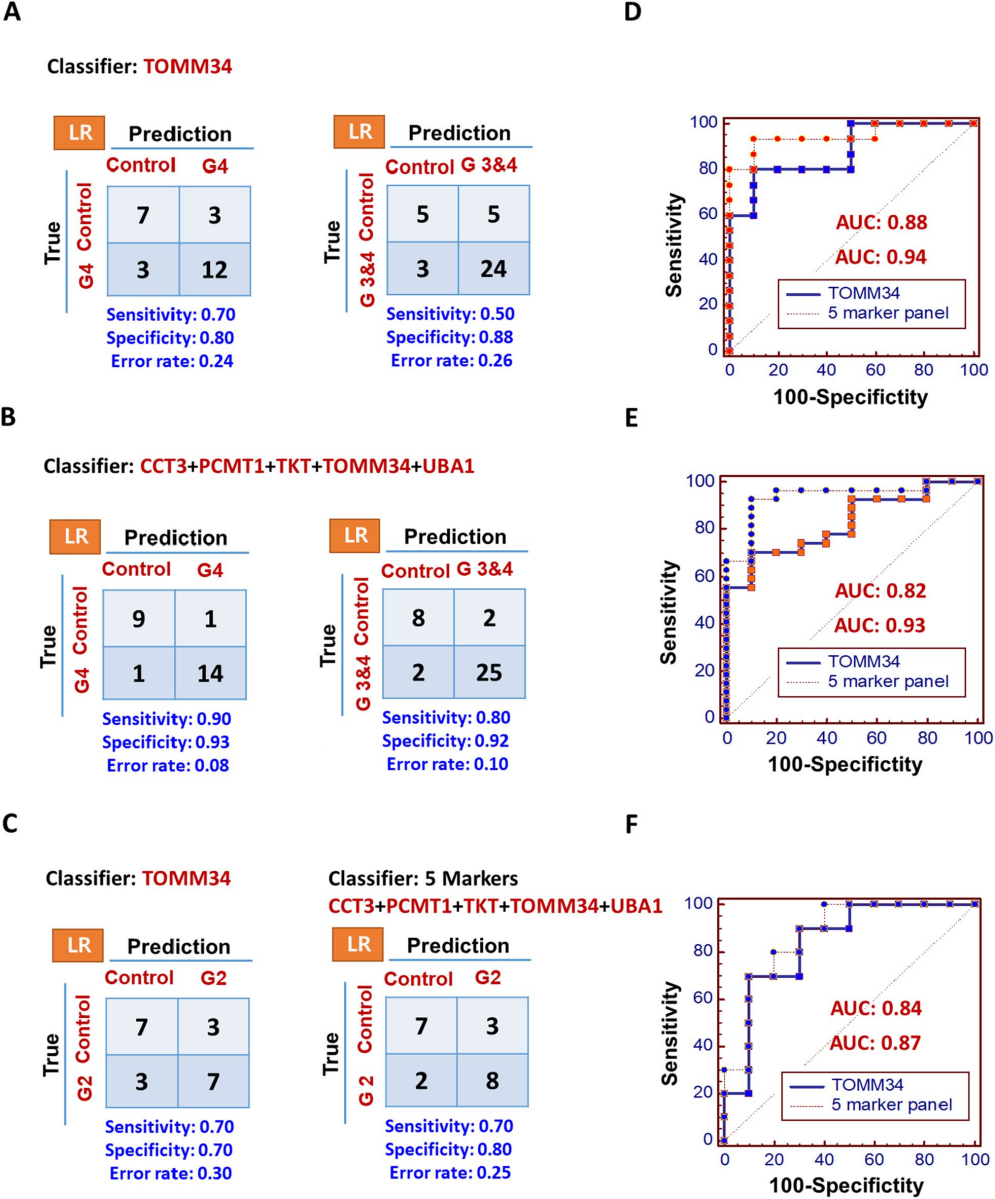
Comparison of discriminatory power of the 5-marker panel versus the best single marker in primary glial cells. Five proteins were selected from *t*-test and ROC curve and used to construct the 5-marker panel, and its performance was evaluated. Logistic regression algorithms were used, in which enter method was used to evaluate the discriminatory power between control and grade 2, 4, 3 and 4 groups (Control: 10, Grade 2: 10, Grade 3: 12, and Grade 4: 15). The results of the evaluation between the best single marker **A** and **C** and 5-marker panel **B** and **C** are presented as confusion matrices with sensitivity, specificity, and error rate, and ROC curves **D**, **E**, and **F** are also represented by AUC values

**Fig. 5:**
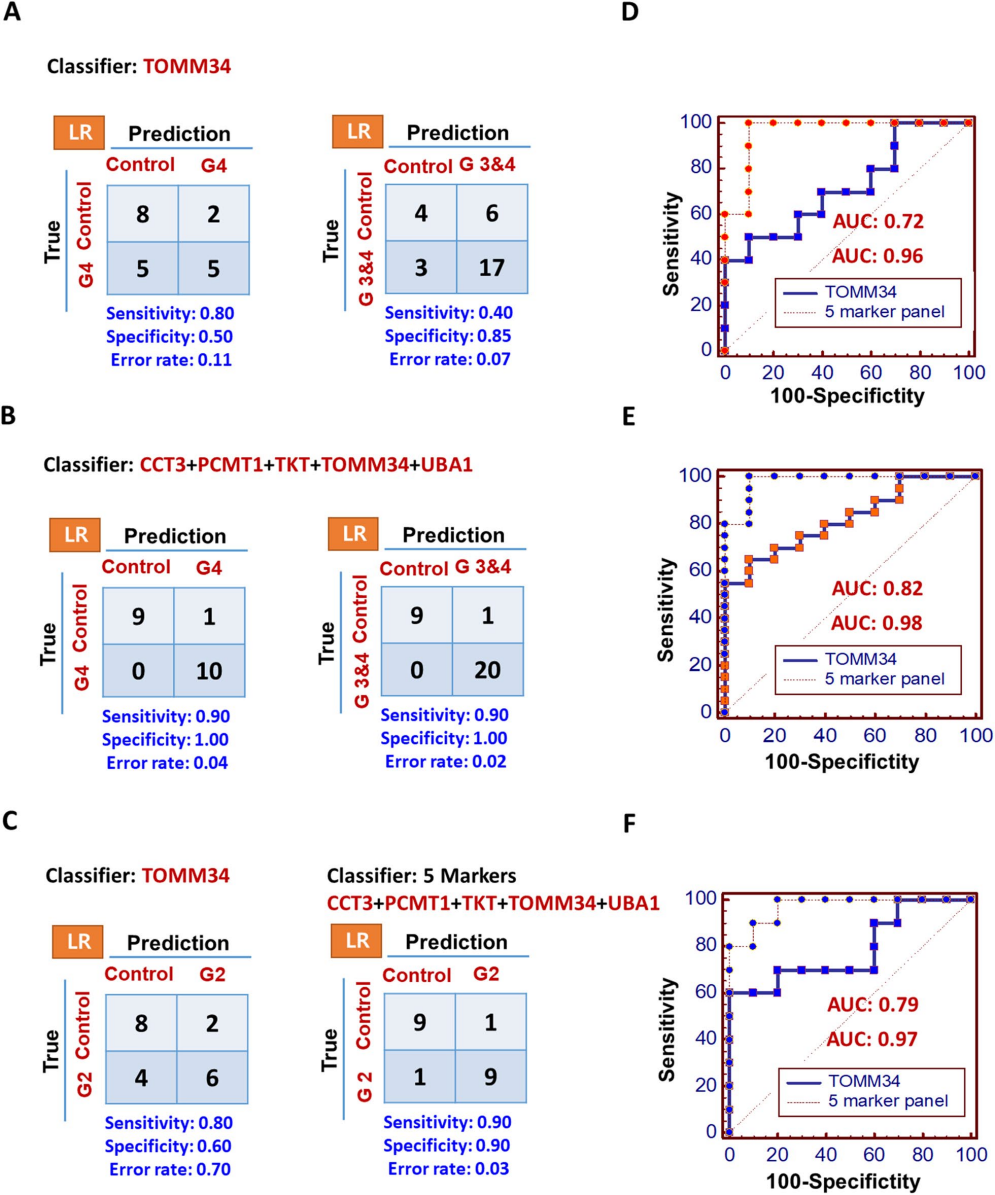
Comparison of discriminatory power of the 5-marker panel versus the best single marker in glial tissues. The performance of 5-marker panel was also evaluated in glial tissue samples. Logistic regression algorithms were used, in which enter method was used to evaluate the discriminatory power between control and grade 2, 4, 3 and 4 groups (Control: 10, Grade 2: 10, Grade 3: 10, and Grade 4: 10). The results of the evaluation between the best single marker **A** and **C** and 5-marker panel **B** and **C** are presented as confusion matrices with sensitivity, specificity, and error rate, and RO**C** curves **D**, **E**, and **F** are also represented by AUC values

In a comparison of the control with cancer group (grade 4), the 5-marker panel (CCT3, PCMT1, TKT, TOMM34, UBA1) showed better sensitivity (0.90 and 0.90), specificity (0.93 and 1.00), error rate (8 and 4%), and AUC value (0.94 and 0.96) than the best single marker (TOMM34). Indeed, the single best candidate model showed lower sensitivity (0.70 and 0.80), specificity (0.80 and 0.50), AUC value (0.88 and 0.72), and a higher error rate (24 and 11%) (Figs. 4, 5 and Additional file 2: Fig. S3). Moreover, for the control versus cancer group (grade 3 and 4) comparison, the 5-marker panel (sensitivity, 0.80 and 0.90; specificity, 0.92 and 1.00; error rate, 10 and 2%; and AUC, 0.93 and 0.98) also showed better performance than the best single marker (sensitivity, 0.50 and 0.40; specificity, 0.88 and 0.85; error rate, 26 and 7%; and AUC, 0.82 and 0.82) (Figs. 4, 5).

## Discussion

To improve the classification discriminating power between the control and cancer groups, we constructed a multi-marker panel and subjected it to statistical evaluation. A similar approach has been conducted to identify a novel biomarker that can distinguish disease status between affected and healthy groups; a multi-marker panel that included more than 1 protein showed better performance than a single marker [29]. Before we selected marker candidates for the multi-marker panel, we first considered which combination of control and cancer groups (grade 2, grade 3, and grade 4) would show the best discriminating power. Grade 2 cancer is an early stage of glioma and it was not easy to observe differences in the control versus cancer group. However, grade 4 cancer is a more advanced stage of disease and may be more representative of a glioma diagnosis than early stage of one. Thus, the detection of grade 4 might be more suitable for glioma screening. Therefore, we first selected a multi-marker panel that showed the best discriminatory power between the control and cancer group (grade 4). We then applied this multi-marker panel to evaluate its discriminatory power in control versus cancer (grade 3 and 4). As shown the result section, the 5-marker panel (sensitivity, 0.80 and 0.90; specificity, 0.92 and 1.00; error rate, 10 and 2%; and AUC, 0.93 and 0.98) also showed better performance than the best single marker (sensitivity, 0.50 and 0.40; specificity, 0.88 and 0.85; error rate, 26 and 7%; and AUC, 0.82 and 0.82) (Figs. 4, 5 and Additional file 2: Fig. S3). These data demonstrate that the discriminatory power of the 5-marker panel was higher than the best single marker model in both of primary cells and tissue (Figs. 4, 5 and Additional file 2: Figure S3).

Furthermore, in this study, we first wanted to know that the discovered single and multi-marker are able to show the classification discriminating power between the control and cancer groups (grade 2) and if so, these markers are also able to show whether or not multi-marker panel represent the better performance than single marker in the control and cancer groups (grade 2). In a comparison of the control with cancer group (grade 2), single best candidate model showed the effective classification discriminating power and the 5-marker panel also showed better sensitivity (0.70 and 0.90), specificity (0.80 and 0.90), error rate (25 and 3%), and AUC value (0.87 and 0.97) than the best single marker (TOMM34) (Fig. 5). Indeed, the single best candidate model showed lower sensitivity (0.70 and 0.80), specificity (0.70 and 0.60), AUC value (0.84 and 0.97), and a higher error rate (30 and 70%) in both of primary cells and tissue (Figs. 4, 5). Consequently, discovered 5 multi-marker showed the classification discriminating power between the control and cancer groups (grade 2).

**Fig. 6:**
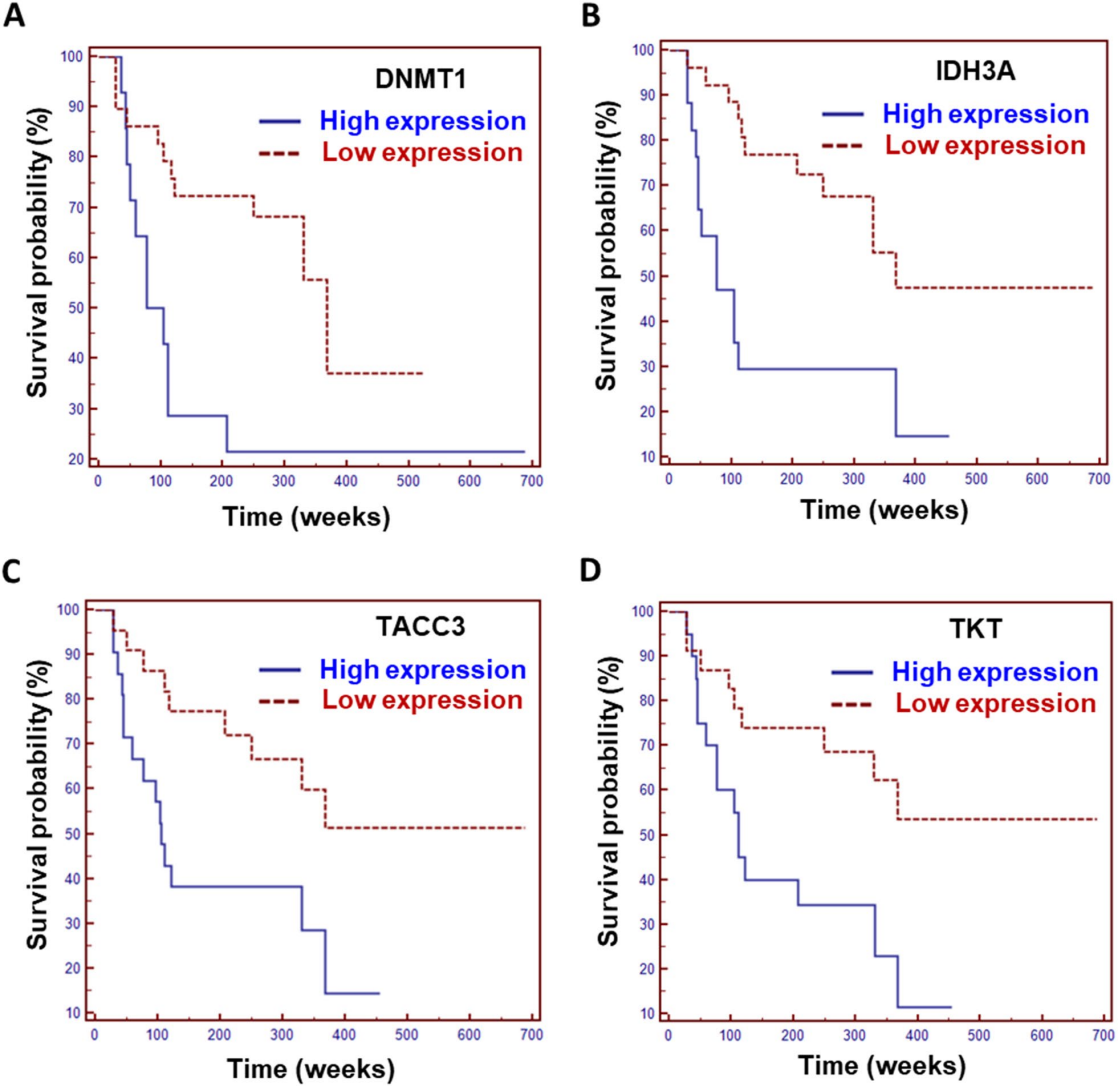
The result of Kaplan–Meier Survival Analysis for DNMT1, IDH3A, TACC3, and TKT For the discovery of prognostic markers, Kaplan–Meier survival curves were generated and compared the protein expression and survival rate. Four proteins (**A** DNMT1, **B** IDH3A, **C** TACC3, and **D** TKT) showed significant differences between the high and low expressions, where p-values of analysis are 0.0226, 0.0033, 0.0130, and 0.0092, respectively

For the discovery of prognostic markers, we used the individual patient’s clinical information, and analyzed the statistical significance between the survival dataset and expression of selected 20 proteins. To this end, Kaplan–Meier survival curves were generated and compared the protein expression and survival rate. We divided the MRM data into two groups, i.e., high and low expressions. In the result of the Kaplan–Meier survival plot, four proteins (DNMT1, IDH3A, TACC3, and TKT) showed significant differences between the high and low expressions, although they did not differ significantly in the t-test. As shown by the Kaplan–Meier plot analysis of the MRM data, we showed that higher expression of these proteins was correlated with poorer prognosis of glioma patients (Fig. 6). This demonstrated that DNMT1, IDH3A, TACC3, and TKT could be incorporated as prognostic markers for glioma. However, 4 prognostic marker candidates seem to be required further validation in a large sample size.

## Conclusion

In this study, 7429 and 476 proteins were identified and quantitated in the control and cancer samples, respectively. Among them, 20 proteins were selected and validated using the quantitative MRM-MS assay, whereas we verified the reproducibility of the overall process for the MRM-MS assay. From the results of the quantitative assay, we discovered five potential diagnostic and four prognostic biomarkers for glioma. The results of this study indicate that our MRM-MS assay has the advantages of being highly validated, transferable, and able to quantify high- to low-abundance proteins, and has the potential for use as a preclinical validation method. Although we acknowledge that the model requires further validation in a large sample size, the five diagnostic and four prognostic biomarkers can be used as baseline data for the development of new therapeutic strategies for glioma.

### Supplementary Information

**Additional file 1: Table S1.** Clinical sample information used in this study. **Table S2.** List of total identified proteins. **Table S3.** List of all quantifiable proteins

**Additional file 2: Figure S1.** Representative primary Cell Line of the control and Glioma. The primary cell line of the control **A** and the Grade 4 glioma **B** were cultured in DMEM media (Welgene) supplemented with 10% fetal bovine serum (FBS; Gibco Invitrogen), 100 U/mL of penicillin, and 100 mg/mL of streptomycin (Gibco Invitrogen) at 37 °C in an atmosphere of 5% CO2 in air. **Figure S2.** Results of Gene Ontology Analysis. The 7739 identified proteins were enriched to Biological process **A**, Molecular functions **B** and Cellular component **C** represent a biological function involving gene or gene product. **Figure S3.** Validation of 5-marker panel in tissue samples (control group and cancer group). Proteins of 5-marker panel (CCT3, PCMT1, TKT, TOMM34, UBA1) were validated by MRM in control (N=10) and cancer (grade 3& grade 4) (N=20) tissue samples.

## Data Availability

All data and materials are available from the authors upon request.

## Abbreviations

CC: Cellular component
BP: Biological process
FDR: False discovery rate
GBM: Glioblastoma multiforme
GO: Gene ontology
IPA: Ingenuity pathway analysis
MF: Molecular function
MRM: Multiple reaction monitoring
ROC curve: Receiver operating characteristic curves
SIS: Stable isotope standards
TMT: Tandem mass tag

## Declarations

**Ethics approval and consent to participate**

The Ethics Committees of the Seoul National University Hospital and New Drug Development Center have been informed and have given their consent to this study. This study was approved by the Institutional Review Board (IRB) of Seoul National University Hospital (IRB approval H-0507–509-153).
