# Correction: Glial cell proteome using targeted quantitative methods for potential multi-diagnostic biomarkers

**DOI:** 10.1186/s12014-024-09456-x

**Published:** 2024-02-10

**Authors:** Narae Kang, Hyun Jeong Oh, Ji Hye Hong, Hyo Eun Moon, Yona Kim, Hyeon-Jeong Lee, Hophil Min, Hyeonji Park, Sang Hun Lee, Sun Ha Paek, Jonghwa Jin

**Affiliations:** 1 New Drug Development Center, Heungdeok-gu, Chungbuk, Cheongju-si, 28160 Korea; 2 Department of Neurosurgery, Cancer Research Institute and Ischemic/Hypoxic Disease Institute, Seoul National University, 28 Yeongeon-dong, Jongno-gu, Seoul, 03080 Korea; 3 Advanced Institute of Convergence Technology, Seoul National University (SNU), Suwon, 16229 Korea; 4 Department of Molecular Medicine & Biopharmaceutical Sciences, Graduate School of Convergence Science and Technology, Seoul National University, 28 Yeongeon-dong, Jongno-gu, Seoul, 03080 Korea; 5 Doping Control Center, Korea Institute of Science and Technology, Hwarang-ro 14-gil, Seongbuk-gu, Seoul, 02792 Korea; 6 School of Mechanical Engineering, Korea University, Seoul, 024841 Republic of Korea; 7 Institute of Chemical Engineering Convergence Systems, Korea University, Seoul, 02841 Republic of Korea; 8 Department of Chemical and Biological Engineering, Hanbat National University, Daejeon, 34158 Korea

**Correction to: Clinical Proteomics (2023) 20:45**

10.1186/s12014-023-09432-x

In this article [1] the author name Sun Ha Paek was incorrectly written as Sun Ha Peak. The original article has been corrected.
