# Supplementary material for: Glial cell proteome using targeted quantitative methods for potential multi-diagnostic biomarkers
Source: Clin Proteomics. 2023 Oct 24;20:45. doi: 10.1186/s12014-023-09432-x (PMC10598909; doi:10.1186/s12014-023-09432-x)
Supplement: Supplementary file 2 — Additional file 2: Figure S1. Representative primary Cell Line of the control and Glioma. The primary cell line of the control A and the Grade 4 glioma B were cultured in DMEM media (Welgene) supplemented with 10% fetal bovine serum (FBS; Gibco Invitrogen), 100 U/mL of penicillin, and 100 mg/mL of streptomycin (Gibco Invitrogen) at 37 °C in an atmosphere of 5% CO2 in air. Figure S2. Results of Gene Ontology Analysis. The 7739 identified proteins were enriched to Biological process A, Molecular functions B and Cellular component C represent a biological function involving gene or gene product. Figure S3. Validation of 5-marker panel in tissue samples (control group and cancer group). Proteins of 5-marker panel (CCT3, PCMT1, TKT, TOMM34, UBA1) were validated by MRM in control (N=10) and cancer (grade 3& grade 4) (N=20) tissue samples. [file 12014_2023_9432_MOESM2_ESM.docx]

**Additional file figure legends**

**Additional file Figure S1. Representative primary Cell Line of the control and Glioma.**

The primary cell line of the control (A) and the Grade 4 glioma (B) were cultured in DMEM media (Welgene) supplemented with 10% fetal bovine serum (FBS; Gibco Invitrogen), 100 U/mL of penicillin, and 100 mg/mL of streptomycin (Gibco Invitrogen) at 37℃ in an atmosphere of 5% CO2 in air.

**Additional file Figure S2. Results of Gene Ontology Analysis.**

The 7,739 identified proteins were enriched to Biological process (A), Molecular functions (B) and Cellular component (C) represent a biological function involving gene or gene product.

**Additional file Figure S3. Validation of 5-marker panel in tissue samples (control group and cancer group)**

Proteins of 5-marker panel (CCT3, PCMT1, TKT, TOMM34, and UBA1) were validated by MRM in control (N=10) and cancer (grade 3 & grade 4) (N=20) tissue samples.

**Additional file Figure S1.**


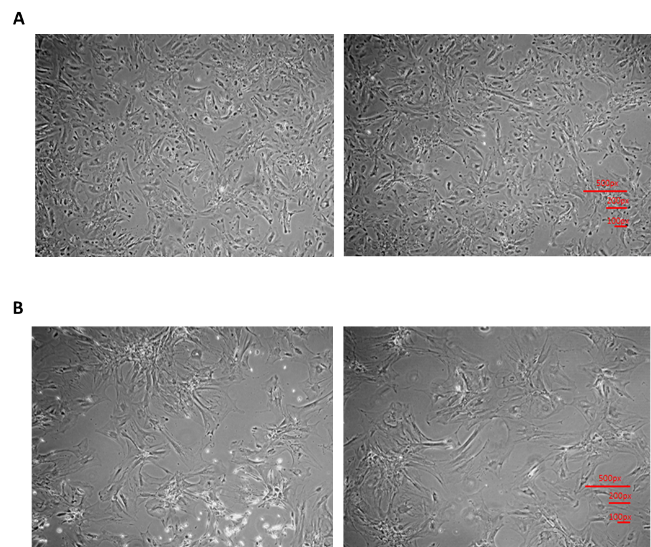


**Additional file Figure S2.**


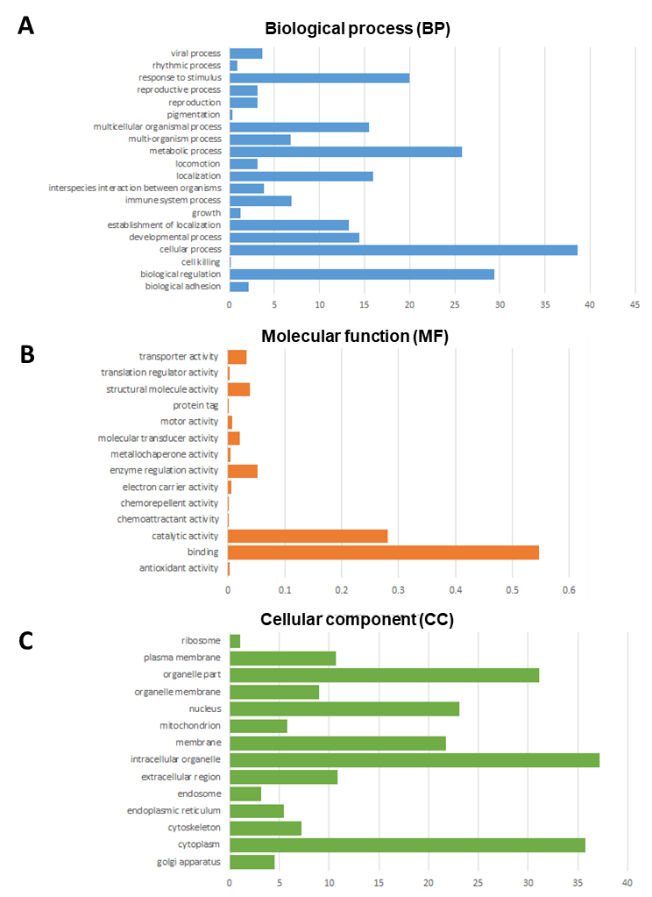


**Additional file Figure S3.**

**
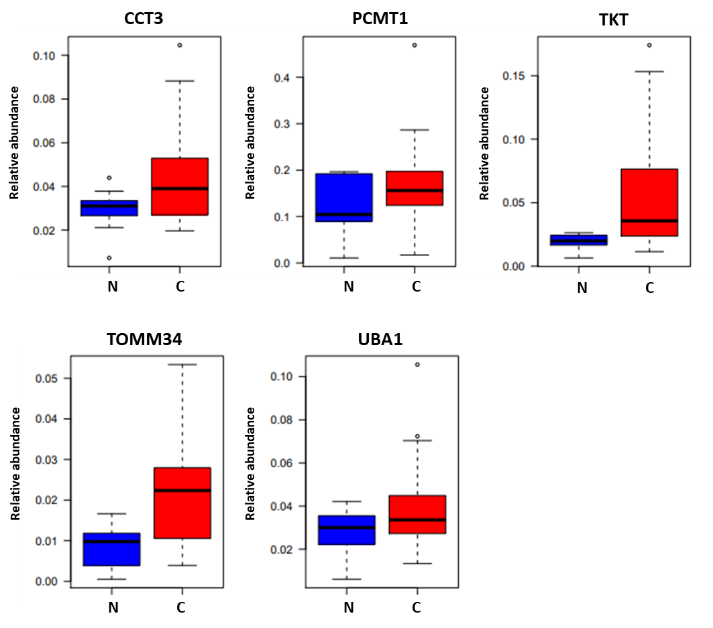
**
